# Intraovarian Platelet-Rich Plasma Injections: Safety and Thoughts on Efficacy Based on a Single Centre Experience With 469 Women

**DOI:** 10.7759/cureus.38674

**Published:** 2023-05-07

**Authors:** Mattheos Fraidakis, Giorgios Giannakakis, Aliki Anifantaki, Meltini Skouradaki, Paraskevi Tsakoumi, Popi Bitzopoulou, Sofia Kourpa, Alexandros Zervakis, Persefoni Kakouri

**Affiliations:** 1 Maternity Unit, Crete Fertility Centre, Heraklion, GRC; 2 Biomedicine Laboratory, Foundation for Research and Technology-Hellas, Heraklion, GRC; 3 Maternity Unit, Crete Fertiltiy Centre, Heraklion, GRC; 4 Microbiology, Mitera Maternity Clinic, Heraklion, GRC

**Keywords:** infertility, menopause, ovarian injection, ovarian rejuvenation, platelet-rich plasma

## Abstract

Background: Ovarian rejuvenation is an innovative procedure intended to restore ovarian fertility and development during the climacteric and has been used to enhance fertility in women with premature ovarian insufficiency (POI). This retrospective study was conducted to determine the effects of an intraovarian platelet-rich plasma (PRP) injection on ovarian stimulation outcomes in women referred to an in vitro fertilisation centre.

Methods-Population: This was a retrospective observational study, and the inclusion criteria included women of reproductive age with at least one ovary with a history of infertility, hormonal abnormalities, an absence of a menstrual cycle, and premature ovarian failure. During the patient's first consultation, a detailed reproductive history was recorded, a pelvic scan for ovarian size was conducted, and hormonal analysis for *follicle-stimulating hormone (*FSH), anti-Müllerian hormone (AMH), estradiol (E2), and luteinizing hormone (LH) was conducted.

Results: In the study, 469 women with a history of infertility, hormonal abnormalities, an absence of a menstrual cycle, and premature ovarian failure had hormonal levels recorded up to four months after treatment, and these were included in the study. The volume of peripheral blood required to prepare 6-8 mL of PRP for administration was 40-60 mL. The initial concentration of platelets in the peripheral blood sample was about 25000/µL, whereas the prepared PRP had a concentration of 900.000/µL. A volume of approximately 2-4 mL per ovary, depending on the ovarian volume, was used for the intraovarian injection.

PRP intervention had significant effects on FSH concentration at the α = 0.05 level. Statistically significant increases in normal values of FSH and E2were observed for months three and four after the PRP intervention for all age groups.

Conclusions: The results of our observational study revealed that a PRP intraovarian injection is associated with improved ovarian tissue and function. Future randomised clinical trials are needed to shed light on the use of PRP in ovarian rejuvenation before offering it routinely in clinical practice.

## Introduction

Because an increasing number of modern women postpone pregnancy until later in life, restoring ovarian function is crucial. Poor ovarian reserve (POR) is one of the main contributing factors to infertility in women of advanced reproductive age. Although these women undergo in vitro fertilisation (IVF) and other infertility interventions, their pregnancy rates remain low, and they have high rates of recurrent pregnancy loss. Anti-müllerian hormone (AMH) and antral follicle count (AFC) are the most sensitive markers to assess ovarian reserve. Several studies have evaluated autologous platelet-rich plasma (PRP) and its effects on infertility.

Platelet-rich plasma (PRP) is becoming popular as a nonoperative treatment option for a broad spectrum of gynaecological disorders [1], particularly infertility. Ovarian rejuvenation is an innovative procedure for restoring ovarian fertility and development during menopause and has been used to enhance fertility in women with premature ovarian insufficiency (POI). The use of PRP for ovarian rejuvenation was first outlined a few years ago in Greece when a group of poor-prognosis infertility patients received an intraovarian injection of PRP followed by in vitro fertilisation (IVF) with their own oocytes [2].

The effectiveness of PRP injections into the ovaries for improving ovarian function has remained subject to speculation. Platelet-rich plasma (PRP) is derived from whole blood, which contains plasma (55%), red blood cells (41%), platelets, and white blood cells (4%), by centrifugation and separation of its different components. The centrifugation and separation processes lead to the removal of red blood cells and the production of plasma with a five- to 10-fold higher concentration of growth factors. Thus, PRP is a natural product containing highly concentrated platelets with growth factors in concentrations that are three to five times higher than plasma [2]. PRP, as an autologous and highly concentrated solution of plasma, is prepared from the patient’s blood and contains a concentrated source of insulin-like growth factors 1 and 2, fibroblast growth factor, epidermal growth factor, transforming growth factor beta, hormones, and cytokines [3,4]. In addition to other growth factors, platelets contain other substances, such as fibronectin, vitronectin, and sphingosine 1-phosphate, that initiate wound healing [1, 5, 6]. Growth factors promote wound healing by initiating the following stages: tissue necrosis resolution, chemotaxis, cell regeneration, cell proliferation and migration, extracellular matrix synthesis, remodelling, angiogenesis, and epithelialization [1, 7].

Considering the angiogenic composition of the ovary and the pivotal influence of platelet-derived growth factors on vascular activation and stabilisation, treatment with autologous PRP may be viewed as an enabler of ovarian tissue regeneration [8]. PRP also contains a member of the TGF-b superfamily, growth differentiation factor 9 (GDF9) [9]. GDF9 is regarded as a biomarker of oocyte maturation potential [10, 11], and its mutations have been linked to premature ovarian dysfunction (4). However, even if effects are seen after PRP injection, it remains possible that simple mechanical disruption rather than growth factors could be responsible for the cases of observed follicular activation [12]. Furthermore, any observed effect may only be temporary.

Several investigators have reported improved responses to ovulation induction after treatment with PRP. However, previously published reports have involved, at most, small case series. Whether PRP actually improves ovarian performance is, therefore, still unknown. PRP is nevertheless widely offered as an established fertility treatment, often under the term "ovarian rejuvenation". Thus, this retrospective study was conducted to determine the effects of intraovarian PRP injection on ovarian stimulation outcomes in a large cohort of women who had been referred to an IVF centre.

## Materials and methods

The selection of patients, procedures, and further monitoring were carried out in the private medical centre Crete Fertility Centre, Heraklion, Crete, Greece. Before being included in the study, the women signed an informed consent form for the procedure and their inclusion in the study. The participants’ hormones, follicle-stimulating hormone (FSH) and estradiol (E2), were monitored at three temporal points (before PRP therapy, at the third, and at the fourth month after the PRP therapy). The women were tested for pregnancy, resulting in negative results, and did not have a medical history of a chronic condition, malignancy, or mental illness. Inclusion criteria included women of reproductive age with at least one ovary and a history of infertility, hormonal abnormalities, an absence of a menstrual cycle, and premature ovarian failure. During the patient’s first consultation, a detailed reproductive history was recorded, a pelvic scan for ovarian size was conducted, and a hormonal analysis for FSH, AMH, E2, and luteinizing hormone (LH) was conducted. In the case of amenorrhea, the hormone evaluation was obtained independently of a menstrual cycle. The FSH, E2, LH, and AMH levels were determined on an unspecified day before initiating the PRP treatment. The option of a PRP treatment was presented to the patient based on the published literature [2, 13].

Exclusion criteria included a current or previous IgA deficiency, ovarian insufficiency secondary to a sex chromosome abnormality, prior major lower abdominal surgery resulting in pelvic adhesions, anticoagulant use for which plasma infusion is contraindicated, a mental health disorder including active substance abuse or dependence, and ongoing malignancy or chronic pelvic pain [14, 15].

PRP was prepared by centrifugation using a T-Lab autologous platelet-rich plasma kit. The volume of peripheral blood required to prepare 6-8 mL of PRP for administration was 40-60 mL. For each patient, blood was collected under sterile conditions, and the tubes were centrifuged at 830 g for eight minutes. Afterwards, a 16-gauge needle connected to a 5 ml syringe was inserted into the tube and advanced to the buffy coat layer. The PRP was drawn up with the syringe without removing the blood clot rich in growth factors. Approximately 2-4 cc of PRP was collected from the first tube, and the second tube was processed in a similar way for a total of 4-8 cc of PRP. The collected PRP solution was transferred to a separate tube and shaken gently for 30-60 seconds. The initial concentration of platelets in the peripheral blood sample was 25,000/µL, whereas the prepared PRP had a concentration of 900.000/µL. The intraovarian injection was performed in the operating room under conscious sedation within two hours of PRP preparation. A volume of approximately 2-4 mL per ovary, depending on the ovarian volume, was used for the intraovarian injection. The essential parts of the technique consisted of a nonsurgical, transvaginal, ultrasound-guided, multifocal needle injection and diffusion in the subcortical layers. The injection included multiple sites, with two to three punctures being performed per ovary using a 17-gauge needle. After the activated PRP injection was bilaterally completed, a careful ultrasound assessment of the pelvis was performed to observe vascular integrity and determine the absence of free pelvic fluid. Sedation with propofol was used for the ovarian PRP injection. For all patients, the procedure was completed in less than seven minutes. Following the procedure, each patient was asked to remain supine and rest for 15 minutes; vital signs were rechecked before home discharge.

Hormone levels following PRP treatment were determined on the second or third day of the subsequent menstrual cycles. The participants had regular follow-ups and blood tests at regular time points following the procedure to study the effects of the proposed treatment on the measurements of the hormones FSH and E2. In the case of amenorrhea, hormone levels were tested every 30 to 40 days, with normal values being FSH of 6.2-10 mIU/ml and E2 of 30-60 pg/ml. The improvement percentage modifying from non-normal (ED2) to normal values (i.e., the difference in the FSH and E2 concentrations before and after the PRP intervention) was calculated as:

FSH% = #{FSHafterPRP ÎFSHnormal ÙFSHbeforePRP ÏFSHnormal}×100 N 

E2% = #{E2afterPRP ÎE2normal ÙE2beforePRP ÏE2normal}×100 N 

For the statistical analysis, the data were analysed with a 3 x 2 Friedman’s two-way analysis of variance (ANOVA) test (three temporal points x two groups as the between-subjects factor) for each age group. FSH and E2 values were not normally distributed at each time point, as assessed by Shapiro-Wilk’s test (p < 0.05). 

## Results

Out of 3,480 women who had undergone PRP treatment in the centre between January 2019 and February 2022, the current study included 469 women (469/3480, 13.5%). The study was a pilot trial that was conducted between April and July 2022 at the AAA Centre of the Crete Fertility Centre, Heraklion, Crete, Greece. It included 469 women participants with an average age of 41.9±4.3 years at the start of therapy. The data were divided into three age groups (32-37, 38-42, and 43-46 years). The number of participants in the 32-37 age group was 80, and in the 38-42 age group, it was 170. The 43-46 age group had 136 patients, and the above-46 age group had 83 patients. The participants’ hormones FSH and E2 were monitored at three temporal points (before PRP therapy, at the third, and at the fourth month after the PRP therapy).

FSH concentration was statistically significantly different at different time points (ED1) during the PRP therapy intervention for all age groups (Table 1).

**Table 1:** The improvement percentage of FSH, E2, and pregnancy percentage

Age range (in years)	FSH (%)		E2 (%)		Pregnancy percentage (%)
	Three months after PRP therapy	Four months after PRP therapy	Three months after PRP therapy	Four months after PRP therapy	
32-37	44.9	59.0	50.0	75.6	25.6
38-42	45.3	64.1	42.4	72.9	27.7
43-46	41.2	60.3	48.5	65.4	24.3

Specifically, for the age range of 32-37 years, FSH concentration was statistically significantly reduced after the PRP intervention (χ2(2) = 37.748, p < 0.001). Pairwise comparisons were performed with a Bonferroni correction for multiple comparisons, and the FSH concentration was statistically significantly different among all three time point pairs (pre-third month (p < 0.001), pre-fourth month (p < 0.001) and the third month to fourth month (p < 0.001)).

For the age range of 38-42 years, FSH concentration was statistically significantly reduced after the PRP intervention (χ2(2) = 40.472, p < 0.001). Pairwise comparisons were performed with a Bonferroni correction for multiple comparisons, and the FSH concentration was statistically significantly different among all three time point pairs (pre-third month (p = 0.001 < 0.05), pre-fourth month (p < 0.001) and the third month to fourth month (p = 0.013 < 0.05)).

For the age range of 43-46 years, FSH concentration was statistically significantly reduced after the PRP intervention (χ2(2) = 50.970, p < 0.001). Pairwise comparisons were performed with a Bonferroni correction for multiple comparisons, and the FSH concentration was statistically significantly different among all three time point pairs (pre-third month (p = 0.001 < 0.05), pre-fourth month (p < 0.001) and the third month to fourth month (p = 0.002 < 0.05).

However, there were no statistically significant differences in E2 concentration at different time points during the PRP therapy intervention for all age groups, namely for the age range of 32-37 years (χ2(2) = 2641, p = 0.267), the age range of 38-42 years (χ2(2) = 1.243, p = 0.537) and the age range of 43-46 years (χ2(2) = 1.933, p = 0.380).

The improvement from non-normal to normal FSH values for the age range of 32-37 years in the fourth month of intervention is depicted in Table 2.

**Table 2:** Normal and non-normal FSH concentrations before and after four months of PRP therapy for the age range of 32–37 years

Before and after four months: cross tabulation					
			After four months		Total
			Non-normal FSH	Normal FSH	
Before	Non-normal FSH	Count	24	46	70
		% of Total	30.8%	59.0%	89.7%
	Normal FSH	Count	3	5	8
		% of Total	3.8%	6.4%	10.3%
Total		Count	27	51	78
		% of Total	34.6%	65.4%	100.0%

McNemar’s test was employed to determine if there was a difference in the proportion of normal FSH and E2 preintervention and postintervention with PRP. The proportion of normal FSH concentrations increased from a preintervention value of 8 to a postintervention value of 51, yielding a statistically significant difference (χ2(1) = 25.29, p< 0.001). The proportion of normal E2 concentrations increased from a preintervention value of 13 to a postintervention value of 71, yielding a statistically significant difference (χ2(1) = 54.150, p < 0.001). 

For the age range 38-42 years, McNemar’s test revealed the transition (TM1) from non-normal to normal FSH presented a statistically significant increase (χ2(1) = 80.012, p < 0.001). The transition from non-normal to normal E2 presented a statistically significant increase (χ2(1) = 77.657, p < 0.001). For the age range 43-46, McNemar’s test revealed the transition from non-normal to normal FSH presented a statistically significant increase (χ2(1) = 80.012, p < 0.001). The transition from non-normal to normal E2 presented a statistically significant increase (χ2(1) = 73.287, p < 0.001). 

There were significant main (ED2) effects of PRP intervention (TM3) on FSH concentration at the α = 0.05 level. Statistically significant increases in normal values of FSH and E2 were observed in the third and fourth months after the PRP intervention for all age groups.

## Discussion

In the current study, we examined the influence of intraovarian injection of autologous PRP on the levels of E2 and FSH and pregnancy outcome in women treated with PRP who had a history of infertility, hormonal abnormalities, an absence of a menstrual cycle, and premature ovarian failure in a single centre. To our knowledge, this is the largest cohort in a study evaluating the efficacy of PRP intraοvarian infusion on ovarian rejuvenation. The results of our retrospective observational study revealed that PRP intraovarian infusion could effectively restore ovarian functionality and hormonal profile. The results from the present study confirm the findings of a previous similar study at our centre that included a smaller sample size.

Intraovarian PRP was injected into 469 women. The main reason for including only a small number of women treated (13.5%) was because of COVID-19 restriction measures. Participants could not come in for follow-up tests, and we did not have follow-up data for up to four months. FSH was significantly reduced post-PRP treatment, and the benefit was obvious in all age groups examined.

During our study, follow-ups of all cases were scheduled on the second or third day of the subsequent menstrual cycles consecutively for six months, and hormone levels following PRP treatment were determined. This was reported for all cases within the subsequent calendar month. In the case of amenorrhea, the hormone levels were tested every 30 to 40 days. A decrease in previously high FSH levels was recorded. The decrease in the FSH level was in agreement with the results of a case series report published recently reporting the use of PRP in menopausal and prematurely menopausal women [16] as well as in a case series reporting the use of PRP in poor responders to IVF [2, 13], with one-month post-PRP hormonal levels influenced similarly (i.e., a decrease in FSH levels). Sfakianoudis et al. also studied the role of intraovarian PRP in poor responders. Their study showed a significant reduction in patients’ FSH levels six weeks following the autologous PRP treatment [4]. The positive effect of PRP on ovarian tissue and function may be mirrored by the decrease in FSH, as previously documented, albeit on a case-series level, by other researchers [2, 13, 16].

Pantos et al. studied eight infertile menopausal women (with amenorrhea of 12 to 96 months). In approximately 40% of the women, menstrual cycles were restored within one to three months after the injection, and 18.5% of them experienced the resumption of ovulation cycles with one to five oocytes obtained from the IVF cycles [2]. Sills et al. investigated the effects of the intraovarian injection of activated PRP in four cases in 2018 and observed increased AMH and significantly decreased FSH levels with at least one embryo obtained from the IVF cycles in all patients [15]. However, the precise mechanism of PRP in ovarian rejuvenation is unknown. A proposed hypothesis is that the cell growth factors present in PRP may stimulate the remaining stem cells in the ovaries and thus provide the necessary conditions for the differentiation of those cells to be strengthened [17].

Notably, no adverse side effects were reported by any of our patients, which is in agreement with the current literature [2, 13, 16]. Furthermore, no studies have reported any side effects from the PRP application on the reproductive system [16]. Various studies highlight that PRP growth factors do not present a risk, are nonmutagenic, and are incapable of inducing tumour generation [18, 19].

Despite the strength of this study, which is that it involved the largest cohort of patients that underwent PRP for ovarian rejuvenation, it is limited by several factors, including its retrospective observational nature and the absence of a placebo control group. The present research was an uncontrolled longitudinal study with all patients receiving the same preintervention and postintervention treatment without a control group. Therefore, for the best possible homogeneity in a valid comparison, a control group should be included in future studies. The relatively limited follow-up period may be an additional limiting factor.

## Conclusions

In the largest cohort evaluating the efficacy of PRP intraοvarian infusion on ovarian rejuvenation, FSH levels were found to be significantly reduced post-PRP treatment, the benefit being obvious in all age groups examined. These findings confirmed the results of previous case reports and smaller observational studies.

Data presented herein indicate that autologous intraovarian PRP infusion may restore ovarian function, enabling the reactivation of the folliculogenesis process and the enhancement of the hormonal profile. This may enable the achievement of pregnancy. However, the evidence for the clinical application of intraovarian PRP injection is novel and has not yet been sufficiently elucidated. Future randomised clinical trials are needed to shed light on the use of PRP in ovarian rejuvenation before offering it routinely in clinical practice.
