# Correction: Intraovarian Platelet-Rich Plasma Injections: Safety and Thoughts on Efficacy Based on a Single Centre Experience With 469 Women

**DOI:** 10.7759/cureus.c371

**Published:** 2025-10-30

**Authors:** Mattheos Fraidakis, Giorgios Giannakakis, Aliki Anifantaki, Meltini Skouradaki, Paraskevi Tsakoumi, Popi Bitzopoulou, Sofia Kourpa, Alexandros Zervakis, Persefoni Kakouri

**Affiliations:** 1 Maternity Unit, Crete Fertility Centre, Heraklion, GRC; 2 Biomedicine Laboratory, Foundation for Research and Technology-Hellas, Heraklion, GRC; 3 Maternity Unit, Crete Fertiltiy Centre, Heraklion, GRC; 4 Microbiology, Mitera Maternity Clinic, Heraklion, GRC

This article has been corrected to address the following errors:

Table 1: some FSH, E2 improvement percentages were incorrectly calculated due to an oversight in the initialization of the variables calculating the improvement percent of FSH, E2 of the custom script written for the analysis.

- In row 1, 61.5 has been corrected to 59.0; 80.8 has been corrected to 75.6. 
- In row 2, 67.6 78.2 64.7 84.7 has been corrected to 45.3 64.1 42.4 72.9. 
- In row 3, 108.8 111.0 111.0 115.4 has been corrected to 41.2 60.3 48.5 65.4

Results, paragraph 3: “Specifically, for the age range of 32-36” has been corrected to “Specifically, for the age range of 32-37”.

The authors deeply regret this oversight.

